
JOURNAL INFORMATION
==============================
NLM Title Abbreviation: ScientificWorldJournal
Journal ID: ScientificWorldJournal
NLM Journal ID: 101131163
Journal ID: TSWJ

The Scientific World Journal

ISSN: 2356-6140
EISSN: 1537-744X
Publisher: Wiley

ARTICLE INFORMATION
==============================
PMCID: PMC6084701
PMID: 30147545
DOI: 10.1100/tsw.2001.108
Article ID: 543609
Article version: 1
Subjects: Short Report

Introduction of Telomerase to Ataxia Teleangiectasia Cells Can Extend Shortend Telomere and Bypass Replicative Senescence

Naka Kazu-hito 1
Matsu-ura Akira 1
Tachibana Akira 2
Ishikawa Fuyuki 3
Ikeda Kyoji 1
Motoyama Noboru 1 *
1 Department of Geriatric Research, National Institute for Longevity Sciences, 36-3 Gengo, Morioka, Obu 474-8522, Japan
2 Radiation Biology Center, Kyoto University, Yoshida-Konoe-cho, Sakyo-ku, Kyoto 606-8501, Japan
3 Department of Life Science, Tokyo Institute of Technology, 4259 Nagatsuta Midori-ku, Yokohama 226-8501, Japan
*Noboru Motoyama: motoyama@nils.go.jp
Publication date: 2001
Electronic publication date: 2001 Apr 4
Volume: 1
First page: 70
Last page: 70
Copyright: Copyright © 2001 Kazu-hito Naka et al.
Copyright year: 2001
License: This is an open access article distributed under the Creative Commons Attribution License, which permits unrestricted use, distribution, and reproduction in any medium, provided the original work is properly cited.
License URL: https://creativecommons.org/licenses/by/3.0/

Funding: Ministry of Health, Labour and Welfare H11-chouju-005

==============================
Miami *Nature Biotechnology* Short Reports
*TheScientificWorld* (2001) 1(S3), 70SR
ISSN 1532-2246; DOI 10.1100/TSW.2001.108

### INTRODUCTION OF TELOMERASE TO ATAXIA TELEANGIECTASIA CELLS CAN EXTEND SHORTEND TELOMERE AND BYPASS REPLICATIVE SENESCENCE

Kazu-hito Naka[1], Akira Matsu-ura[1], Akira Tachibana[2], Fuyuki Ishikawa[3], Kyoji Ikeda[1] and Noboru Motoyama[1]*

[1]Department of Geriatric Research, National Institute for Longevity Sciences, 36-3 Gengo, Morioka, Obu 474-8522, Japan, [2]Radiation Biology Center, Kyoto University, Yoshida-Konoe-cho, Sakyo-ku, Kyoto 606-8501, Japan, [3]Department of Life Science, Tokyo Institute of Technology, 4259 Nagatsuta Midori-ku, Yokohama 226-8501, Japan
* motoyama@nils.go.jp

**INTRODUCTION.** Ataxia teleangiectasia (AT) is an autosomal recessive disorder characterized by progressive neurological degeneration, teleangiectasia, high sensitivity to ionizing radiation, high incidence of cancer, and premature aging of skin and hair (1). Cells derived from AT patients exhibit a variety of abnormalities in culture, such as hypersensitivity to ionizing radiation, rapid shortening of telomere length, and premature replicative senescence. Normal diploid fibroblasts in culture have a finite proliferative lifespan and enter a nondividing state termed replicative senescence (2). Ectopic expression of the catalytic subunit of telomerase, hTERT, has been shown to restore telomerase activity, stabilize telomere length and bypass replicative senescence in telomerase-negative normal fibroblasts (3) as well as in the premature aging Werner syndrome (4). In order to address a role of ATM (AT mutated) protein in the regulation of telomere length and a relationship between telomere length and premature replicative senescence in AT cells, we have examined whether fibroblasts from AT patients can be immortalized by introduction of hTERT.

**METHODS.** Primary dermal fibroblasts from normal and AT individuals were used. Amphotrophic retrovirus expressing hTERT was created with pMX-puro-hTERT in Phenix-A cells. Cells were infected with the hTERT retrovirus, and selected in culture containing 0.5 μg / ml puromycin. Telomere length was measured by terminal restriction fragment (TRF) assay, and telomerase activity was assessed by telomeric repeat amplification protocol (TRAP) using TRAP*eze* kit (Intergen, Purchase, NY).

**RESULTS.** Expression of hTERT mRNA and telomerase activity in hTERT-infected AT as well as normal cells was confirmed by RT-PCR and TRAP assays, respectively. Telomere length of hTERT-infected AT cells was extended to approximately 9 kb, compared with short telomeric DNA of about 6kb in the parent AT cells at near mortal 1 (M1) phase. Whereas parental AT cells underwent replicative senescence at about 24 population doublings (PDs), hTERT-positive AT cells survived over 40 PDs, similar to hTERT-infected normal fibroblasts (Fig. 1).

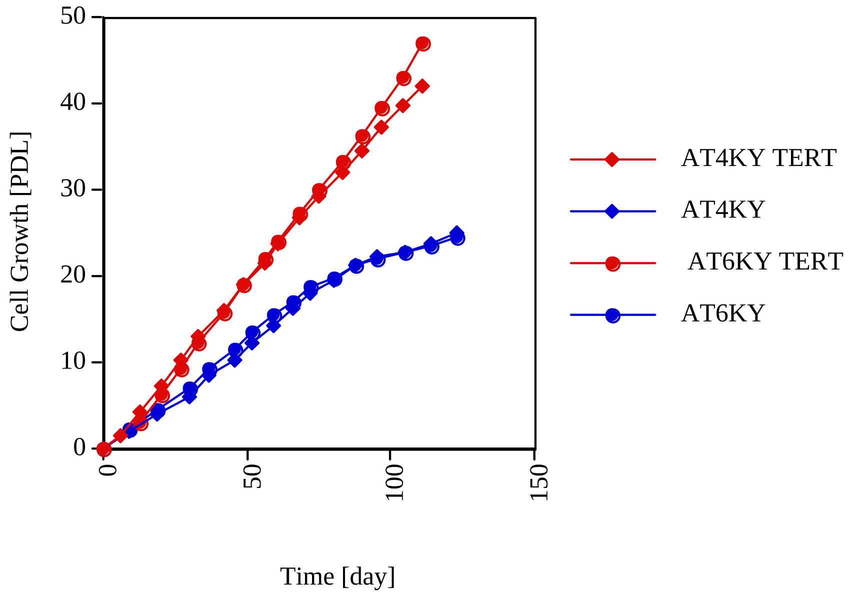

**Fig. 1.** Growth curves of AT and normal fibroblasts in the presence or absence of hTERT transduction.

**DISCUSSION.** The observation that the shortened telomere length can be reversed by introduction of TERT in AT cells suggests that ATM protein may be dispensable for the regulation of telomere length in the presence of telomerase. In addition, since replicative senescence can be rescued by telomere elongation in AT as well as normal fibroblasts, telomere length rather than ATM protein itself may be responsible for the premature senescent phenotype in AT cells.

**ACKNOWLEDGEMENTS.** This work was supported by Health Science Research Grant for Comprehensive Research on Aging and Health (H11-chouju-005 to N.M.) from the Ministry of Health and Welfare of Japan.

**REFERENCES.**

1. Lavin, M.F. and Shiloh, Y. (1997) Annu. Rev. Immunol. 15, 177-202
2. Hayflick, L. and Moorhead, P.S. (1961) Exp. Cell Res. 25, 585-621
3. Bodnar, A.G. et al. (1998) Science 279, 349-352
4. Wyllie, F.S. et al. (2000) Nat. Genet. 24, 16-17
